# *Ex Vivo* VEGF Delivery by Neural Stem Cells Enhances Proliferation of Glial Progenitors, Angiogenesis, and Tissue Sparing after Spinal Cord Injury

**DOI:** 10.1371/journal.pone.0004987

**Published:** 2009-03-25

**Authors:** Hyuk Min Kim, Dong Hoon Hwang, Jong Eun Lee, Seung U. Kim, Byung G. Kim

**Affiliations:** 1 Brain Disease Research Center, Institute for Medical Sciences, Ajou University School of Medicine, Suwon, Korea; 2 Department of Neurology, Ajou University School of Medicine, Suwon, Korea; 3 Department of Anatomy, BK21 Project for Medical Science, Yonsei University College of Medicine, Seoul, Korea; 4 Division of Neurology, Department of Medicine, University of British Columbia, Vancouver, Canada; 5 Medical Research Institute, Chungang University School of Medicine, Seoul, Korea; University of North Dakota, United States of America

## Abstract

The present study was undertaken to examine multifaceted therapeutic effects of vascular endothelial growth factor (VEGF) in a rat spinal cord injury (SCI) model, focusing on its capability to stimulate proliferation of endogenous glial progenitor cells. Neural stem cells (NSCs) can be genetically modified to efficiently transfer therapeutic genes to diseased CNS. We adopted an *ex vivo* approach using immortalized human NSC line (F3 cells) to achieve stable and robust expression of VEGF in the injured spinal cord. Transplantation of NSCs retrovirally transduced to overexpress VEGF (F3.VEGF cells) at 7 days after contusive SCI markedly elevated the amount of VEGF in the injured spinal cord tissue compared to injection of PBS or F3 cells without VEGF. Concomitantly, phosphorylation of VEGF receptor flk-1 increased in F3.VEGF group. Stereological counting of BrdU+ cells revealed that transplantation of F3.VEGF significantly enhanced cellular proliferation at 2 weeks after SCI. The number of proliferating NG2+ glial progenitor cells (NG2+/BrdU+) was also increased by F3.VEGF. Furthermore, transplantation of F3.VEGF increased the number of early proliferating cells that differentiated into mature oligodendrocytes, but not astrocytes, at 6 weeks after SCI. F3.VEGF treatment also increased the density of blood vessels in the injured spinal cord and enhanced tissue sparing. These anatomical results were accompanied by improved BBB locomotor scores. The multifaceted effects of VEGF on endogenous gliogenesis, angiogenesis, and tissue sparing could be utilized to improve functional outcomes following SCI.

## Introduction

Spinal cord injury (SCI) results in severe and permanent disability, yet there is no single effective therapeutic option to improve functional outcomes. Growth factor treatment is considered as one of the important components for the future combinatorial strategies to repair injured spinal cord [1], [2]. Vascular endothelial growth factor (VEGF) was originally characterized as a potent stimulator of angiogenesis. Later, multifaceted trophic effects of VEGF have been uncovered in nervous tissue [3]. VEGF provides direct protective effects on neurons [4], [5] and enhances neurite outgrowth [6]. It also supports survival and proliferation of various glial cells [7], [8]. The neuroprotective effects of VEGF as well as the angiogenic activity led to improved functional outcomes in animal models of traumatic spinal cord injury and other neurological disorders [9]–[11].

Endogenous stem or progenitor cells that can differentiate into neurons and glial cells are also present in adult spinal cord [12]. The progenitors in glial lineage are stimulated to proliferate in response to SCI [13]–[16]. Proliferating glial progenitors are persistently found until several weeks after injury [13], and they are believed to differentiate into mature glial cells, eventually replacing the lost oligodendrocytes and astrocytes [16]. These findings suggest a promising possibility that mobilization of endogenous glial progenitors can provide a therapeutic opportunity to repair the white matter damaged by traumatic SCI. Recently, the versatile actions of VEGF has been expanded to stimulating proliferation of endogenous neural stem or progenitor cells, and VEGF was shown to increase endogenous neurogenesis after stroke [11], [17]. However, potential effects of VEGF on the glial progenitor cells in the spinal cord after injury have not been investigated.

The present study was undertaken to examine multifaceted therapeutic effects of VEGF in a rat model of contusive SCI, focusing on its capability to stimulate proliferation of endogenous glial progenitor cells. Sustained delivery of growth factors to diseased CNS remains a demanding challenge. Engraftment of genetically modified neural stem cells (NSCs) has proved to be an excellent approach to provide various growth factors [18]–[21]. For stable and robust expression of VEGF, we transplanted VEGF overexpressing immortalized neural stem cells (NSCs) into the injured spinal cord. Our data showed that transplantation of VEGF overexpressing NSCs stimulated proliferation of glial progenitor cells and increased the number of newly born oligodendrocytes. We also report that the *ex vivo* delivery of VEGF enhanced angiogenesis and tissue sparing, leading to improved locomotor recovery.

## Results

### *Ex vivo* VEGF delivery to the injured spinal cord using immortalized human NSCs

**Figure 1:**
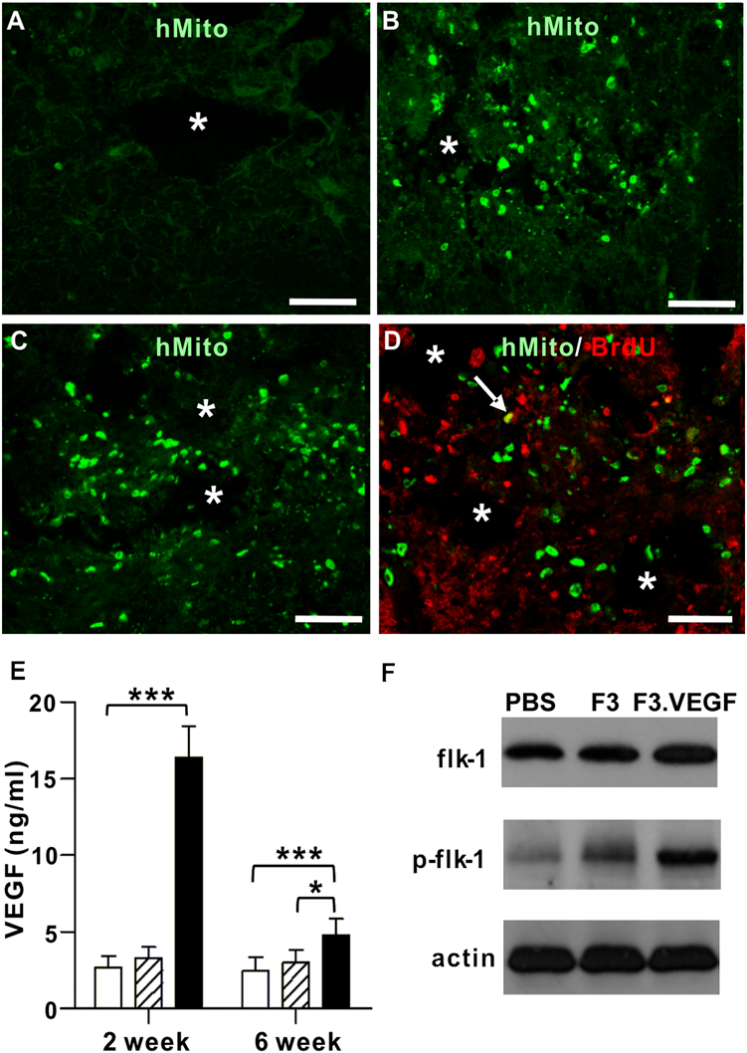
*Ex vivo* delivery of VEGF carried by human neural stem cells (NSCs) to the injured spinal cord. (A–C) Detection of transplanted NSCs by human specific mitochondria (hMito) staining at 1 week after transplantation (2 weeks after injury). Surviving F3 (B) and F3.VEGF (C) cells were observed around the lesion cavities. Control PBS group did not show any immunoreactivity to hMito (A). (D) Most of the transplanted NSCs did not incorporate BrdU. An arrow indicates a transplanted NSC colocalized with BrdU. Asterisks indicate lesion cavities at the epicenter. Scale bars; 100 um. (E) Levels of VEGF production in spinal cord tissue were measured by ELISA at 2 and 6 weeks after injury (1 and 5 weeks after transplantation). White, hatched, and black bars represent PBS (N = 5), F3 (N = 5), and F3.VEGF (N = 5) groups, respectively. * *p*<0.05, *** *p*<0.001 by one-way ANOVA followed by Tukey's *posthoc* analysis. (F) Western blot analysis of VEGF receptor flk-1 and phosphorylated flk-1 (p-flk-1).

One of the immortalized human NSC line (F3) was retrovirally transduced with human VEGF cDNA to generate VEGF overexpressing NSC line (F3.VEGF). At 1 week after SCI, parental F3 NSCs or VEGF overexpressing NSCs (F3.VEGF) were transplanted at 2 mm rostral and caudal to the epicenter. Grafted NSCs were detected by immunoreactivity against human specific mitochondria at 1 week after transplantation (Fig. 1A–C). The number of surviving F3.VEGF cells was higher than that of F3 cells. The percentage of surviving cells versus total transplanted cells was 15.9±4.3 and 28.7±7.6% for F3 and F3.VEGF groups, respectively (N = 5 for each group). The majority of surviving cells were observed at the lesion epicenter, suggesting that grafted cells migrated towards the injury sites. Fewer cells were found to be scattered in distant areas rostral and caudal to the lesion. Most of the grafted NSCs did not incorporate proliferation marker BrdU (Fig. 1D), indicating that they no longer proliferate after being transplanted into the injured spinal cord. Grafted cells were still detected at 5 week post-transplantation, but the number of surviving cells (both F3 and F3.VEGF cells) was markedly reduced by that time point; 9.1±3.3 (F3) and 11.6±4.8% (F3.VEGF) of transplanted cells were detected (N = 5 for each group).

The amount of VEGF in the injured spinal cord tissue was determined by ELISA assay. Transplantation of F3.VEGF cells dramatically upregulated the level of VEGF at 2 weeks after SCI (7 days after transplantation) (2.70±0.724 ng/ml, 3.25±0.78 ng/ml, and 16.40±2.05 ng/ml for PBS, F3, and F3.VEGF groups, respectively; N = 5 for each group) (Fig. 1E). Concomitantly, immunoblot analysis revealed that F3.VEGF grafts increased phosphorylation of VEGF receptor flk-1 (Fig. 1F). In contrast, the expression of flk-1 protein itself was not affected by the *ex vivo* VEGF delivery. The amount of VEGF was still higher in F3.VEGF group until 6 weeks than in PBS or F3 group, but the extent of increase sharply declined compared to that measured at 2 weeks after SCI (2.45±0.89 ng/ml, 2.99±0.81 ng/ml, and 4.77±1.07 ng/ml for PBS, F3, and F3.VEGF groups, respectively).

### Proliferation of glial progenitor cells by F3.VEGF grafts

**Figure 2:**
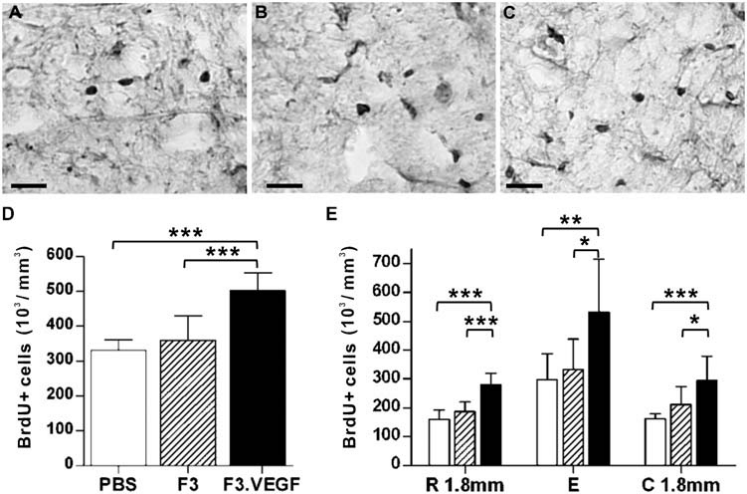
Transplantation of F3.VEGF enhanced cellular proliferation. (A–C) Representative images of BrdU stained sections from PBS (A), F3 (B), and F3.VEGF (C) groups. Scale bars; 50 um. (D) The number of BrdU+ cells per 1 mm^3^ of spinal cord tissue was stereologically counted and compared between the three groups at 2 weeks after injury. (E) The number of BrdU+ cells was separately counted at the epicenter (designated as E) and 1.8 mm rostral and caudal (designated as R and C, respectively) to the epicenter. * *p*<0.05, ** *p*<0.01, *** *p*<0.001 by one-way ANOVA followed by Tukey's *posthoc* analysis. White, hatched, and black bars represent PBS (N = 8), F3 (N = 8), and F3.VEGF (N = 8) groups, respectively.

**Figure 3:**
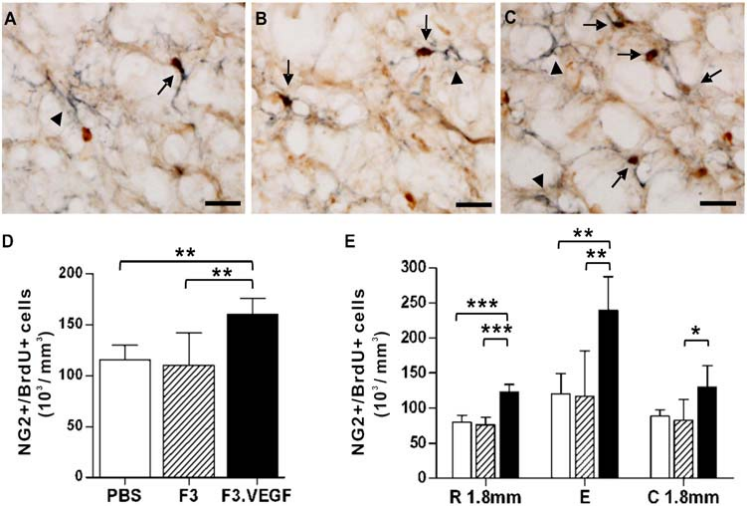
Proliferation of NG2+ glial progenitor cells. (A–C) Representative images of spinal cord sections doubly stained with BrdU (dark brown) and NG2 (dark blue) cells from PBS (A), F3 (B), and F3.VEGF (C) groups. Arrows indicate NG2+/BrdU+ cells and arrow heads NG2 single positive cells. Scale bars; 50 um. (D) The number of NG2+/BrdU+ cells per 1 mm^3^ of spinal cord tissue was stereologically counted and compared between the three groups at 2 weeks after injury. (E) The number of NG2+/BrdU+ cells was separately counted at the epicenter (designated as E) and 1.8 mm rostral and caudal (designated as R and C, respectively) to the epicenter. * *p*<0.05, ** *p*<0.01, *** *p*<0.001 by one-way ANOVA followed by Tukey's *posthoc* analysis. White, hatched, and black bars represent PBS (N = 5), F3 (N = 5), and F3.VEGF (N = 5) groups, respectively.

To determine the effects of *ex vivo* delivery of VEGF on cellular proliferation after SCI, rats received BrdU for three consecutive days before being sacrificed at 2 weeks after SCI (1 week after transplantation). Stereological counting of BrdU+ cells showed that transplantation of VEGF overexpressing NSCs increased the number of proliferating cells compared to the other groups (Fig. 2A–C): Animals with F3.VEGF grafts contained more than 1.5 fold higher number of BrdU+ cells than those with control PBS injection or parental F3 grafts at 2 weeks (*p*<0.001 compared to PBS or F3 group) (Fig. 2D). The effect of F3.VEGF on cellular proliferation was significant across the rostrocaudal regions (Fig. 2E). The cellular proliferation was most active at the epicenter region in all groups, and there was no remarkable difference between the regions 1.8 mm rostral and caudal to the epicenter. We next determined the effects of VEGF on the proliferation of NG2+ glial progenitor cells. About 40% of BrdU+ cells were doubly positive to NG2 across the different experimental groups (Fig. 3A–C). The number of NG2+/BrdU+ cells per 1 mm^3^ of spared tissue was greatly increased by F3.VEGF grafts at 2 weeks compared to PBS or F3 injected group (Fig. 3D) (*p*<0.001 compared to PBS or F3 groups). The rostrocaudal distribution of proliferating NG2+ glial progenitor cells was very similar to that of BrdU+ cells (Fig. 3E). The increase of NG2+/BrdU+ cells by F3.VEGF was most obvious at the epicenter region, and there was no remarkable difference between the regions 1.8 mm rostral and caudal to the epicenter.

### Long-term fate of early proliferating glial progenitor cells

**Figure 4:**
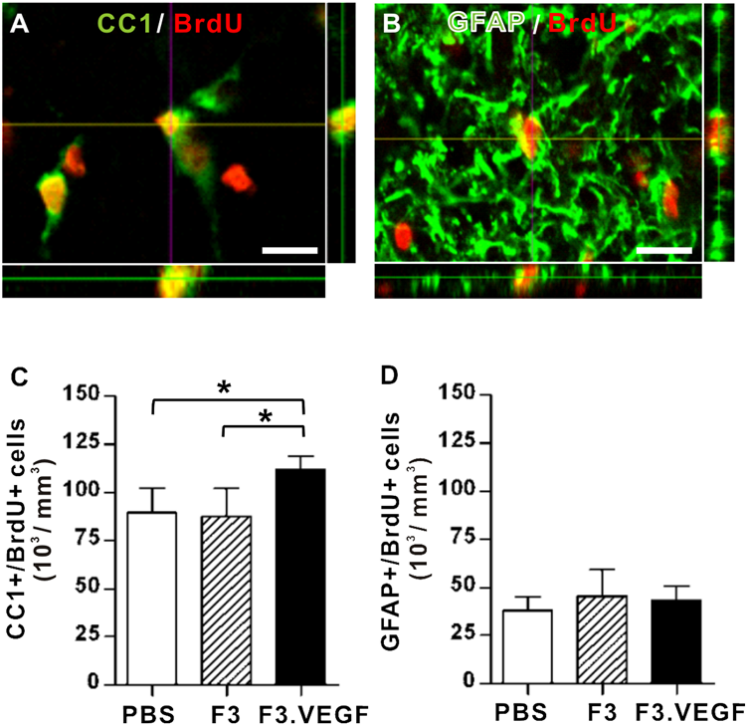
Long term fate of early proliferating glial progenitor cells. (A–B) Confocal images of BrdU incorporated cells colocalized with mature oligodendrocyte marker CC1 (A) and astrocyte marker GFAP (B). Scale bars; 10 um. (C–D) The number of CC1+/BrdU+ (C) and GFAP+/BrdU+ (D) cells per 1 mm^3^ of spinal cord tissue was stereologically counted and compared between the three groups at 6 weeks after injury. * *p*<0.05 by one-way ANOVA followed by Tukey's *posthoc* analysis. N = 5 for each group.

To determine long-term fate of early proliferating glial progenitor cells, BrdU injected animals were allowed to survive 4 more weeks and then the phenotypic differentiation of BrdU+ cells was examined at 6 weeks after SCI. We found that approximately 30% (34.9±1.2%, 29.8±0.9%, and 32.2±1.0% for PBS, F3, and F3.VEGF groups, respectively) of early proliferating cells (BrdU+) differentiated into CC1+ mature oligodendrocytes (Fig. 4A). Some of BrdU+ cells were colocalized with GFAP (Fig. 4B), but their percentage (14.0±1.4%, 14.3±1.0%, and 11.5±0.7% for PBS, F3, and F3.VEGF groups, respectively) was less than that of CC1+/BrdU+ cells. Stereological assessment showed that the number of newly born oligodendrocytes was significantly increased by transplantation of F3.VEGF (*p*<0.05 compared to PBS or F3 group) (Fig. 4C). In contrast, the number of newly born GFAP+ astrocytes was not different between the groups (Fig. 4D). Double labeling for BrdU and the neuron-specific markers (NeuN or MAP2) showed no colocalization (data not shown), indicating that early proliferating cells did not differentiate into neurons regardless of experimental interventions.

### F3.VEGF grafts enhance angiogenesis, tissue sparing, and functional recovery

**Figure 5:**
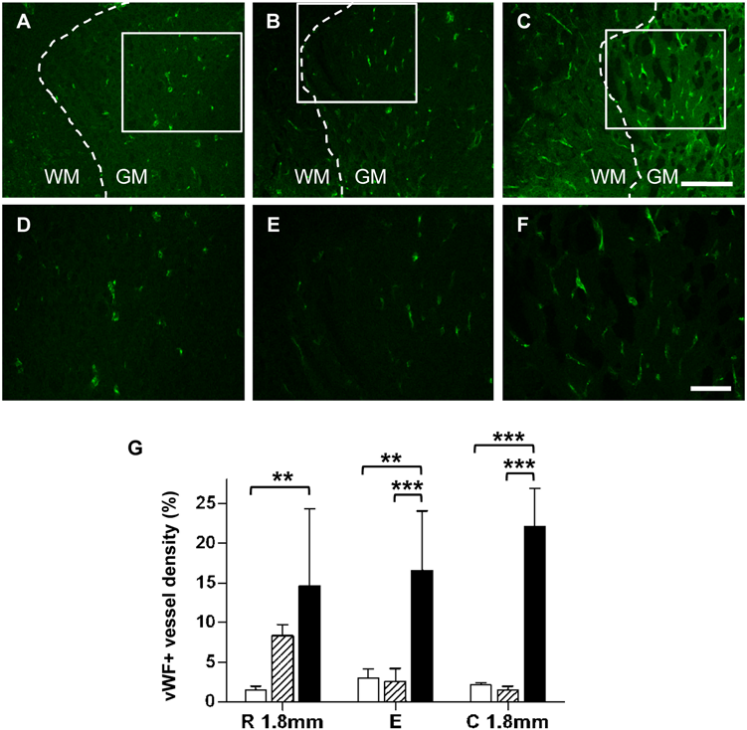
Promotion of angiogenesis by transplantation of F3.VEGF. (A–F) Representative images of spinal cord sections (6 weeks after injury) stained with blood vessel marker vWF from PBS (A, D), F3 (B, E), and F3.VEGF (C, F) groups. Figures in the lower panel (D, E, F) are high power images of the boxed regions in (A, B, C), respectively. Scale bars in the upper panel; 200 um. Scale bars in the lower panel; 50 um. (G) The fraction of areas occupied by vWF immunoreactive vessels was compared between the three groups at the epicenter (designated as E) and 1.8 mm rostral and caudal (designated as R and C, respectively). ** *p*<0.01, *** *p*<0.001 by one-way ANOVA followed by Tukey's *posthoc* analysis. White, hatched, and black bars represent PBS (N = 5), F3 (N = 5), and F3.VEGF (N = 5) groups, respectively.

We determined the extent of angiogenesis by measuring the density of vWF positive microvessels. As seen in Fig. 5, F3.VEGF grafts dramatically (by more than 5-fold compared to PBS group) increased the density of vWF+ vessels in the injured spinal cord, indicating that the extent of angiogenesis was greatly enhanced by the *ex vivo* delivery of VEGF. This angiogenesis-promoting effect by F3.VEGF was evident across the rostrocaudal extent (Fig. 5D). The highest vessel density was observed at the caudal region.

**Figure 6:**
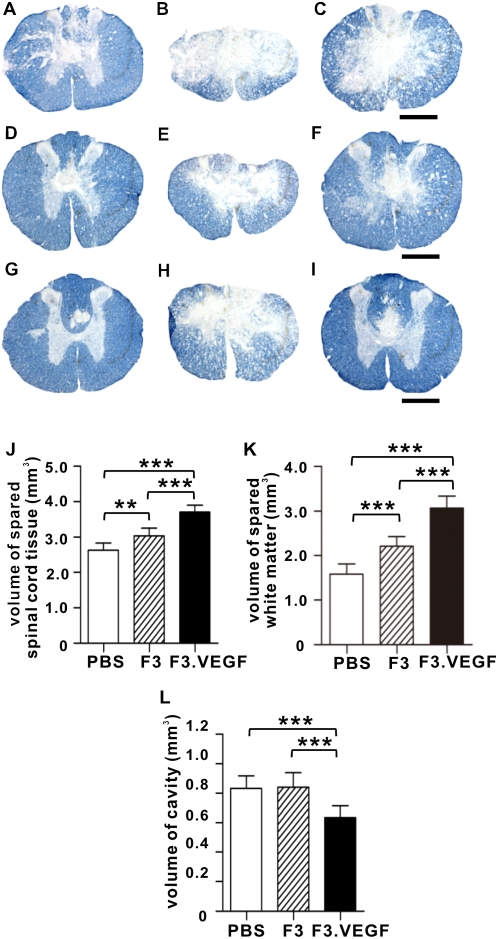
Sparing of spinal cord tissue at 6 weeks after injury. (A–I) Representative images of erichrome stained sections at the 1.8 mm rostral to the epicenter (A, D, G), epicenter (B, E, H), and 1.8 mm caudal to the epicenter (C, F, I). (A–C) PBS, (D–F) F3, and (G–I) F3.VEGF groups. Scale bar; 500 um. (J–L) The volumes of spared spinal cord tissue (J), white matter (K), and lesion cavities (L) were calculated by Cavelieri's method and compared between the three groups. ** *p*<0.01, followed by Tukey's *posthoc* analysis. N = 8 for each group.

SCI usually leaves large central cavities around the epicenter region with varying amount of remaining gray matter and a peripheral rim of spared white matter. Animals with F3.VEGF grafts showed smaller cavitary lesions and a larger amount of spared tissue (Fig. 6A–I). Quantification data showed that transplantation of F3.VEGF significantly increased the volume of spared spinal cord tissue (p<0.001 compared to PBS or F3 group) (Fig. 6J). At the same time, the volume of spared white matter was also increased in F3.VEGF group (p<0.001 compared to PBS or F3 group) (Fig. 6K). This tissue sparing effect was also observed by transplantation of F3 cells. The volume of cystic cavities was reduced by F3.VEGF at 6 weeks after SCI (p<0.001 compared to PBS or F3 group, respectively) (Fig. 6L).

**Figure 7:**
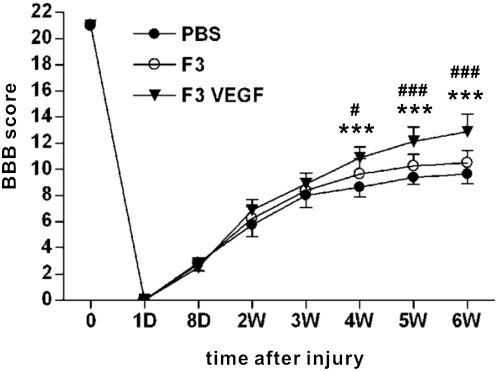
Locomotor recovery assessed by Basso, Beattie, and Bresnahan (BBB) test. Hind limbs locomotor function was scored as from 0 to 21 (flaccid paralysis to normal gait). Rats with transplantation of F3.VEGF showed significantly improved locomotor behavior up to 6 weeks after SCI. *** *p*<0.001 compared to PBS group, ^#^ *p*<0.05, ^###^ *p*<0.001 compared to F3 group, by one-way ANOVA followed by Tukey's *posthoc* analysis. N = 8 for each group.

Locomotor recovery was evaluated using BBB score. The hindlimbs of all the animals in the three groups were completely paralyzed one day after contusion injury, and locomotor function gradually improved thereafter. At 4 weeks after injury (3 weeks after transplantation), the mean score of rats with F3.VEGF grafts was significantly higher than those for the other groups, and remained higher thereafter (Fig. 7). Repeated measures two-way ANOVA revealed a significant treatment effect on locomotor recovery (p<0.001), and one-way ANOVA at each time point showed significant differences between F.VEGF and the other two groups at 4, 5, and 6 weeks after SCI.

## Discussion

The present experiment adopted an *ex vivo* approach for stable and robust expression of VEGF in the injured spinal cord. As expected, transplantation of VEGF overexpressing NSCs elevated the level of VEGF in the injured spinal cord until 6 weeks after SCI. Furthermore, phosphorylation of the VEGF receptor flk-1, which plays a major role in proliferation of precursor cells, angiogenesis, and neuroprotection [3], [17], [22], was markedly enhanced by F3.VEGF grafts. These findings indicate that the *ex vivo* approach using immortalized NSCs ensured a stable and effective increase of the ambient concentration of VEGF in the injured spinal cord, which would be highly demanding or very costly to achieve by direct infusion of VEGF [23]. As gene delivery vehicles, NSCs exhibit inherent long-distance migratory capabilities and a remarkable capacity to integrate with host neural tissue [21]. Especially, immortalized human NSCs have shown exceptional capability to find pathological regions [24]. The majority of F3.VEGF NSCs in this study were also found around the lesion cavities, even though they were injected at 2 mm rostral and caudal to the epicenter. Thus, it is highly likely that F3.VEGF grafts functioned as localized and sustained cellular sources providing VEGF directly to the lesion site.

The major finding of this study was that F3.VEGF grafts markedly increased the number of BrdU+ proliferating cells. Approximately 40% of all the proliferating cells were NG2+ cells in all the groups. This percentage is comparable to the data of the previous report that almost half of the acutely dividing cells were NG2 immunoreactive [14]. Other proliferating cells after SCI are thought to encompass macrophages/microglial cells, Schwann cells, mature glial cells, ependymal cells, fibroblasts, and endothelial cells [13], [16], [25]. It is likely that the *ex vivo* delivery of VEGF promoted proliferation of these potentially proliferating cells to a similar extent. Indeed, the mitogenic role of VEGF has been demonstrated for very different kinds of neural cells. For examples, application of VEGF increased the number of neuronal cells in developing retina [26], and VEGF promoted proliferation of astrocytes [27], Schwann cells [8], and neural stem or progenitor cells [17]. In contrast to our present results, a previous study reported that a single injection of VEGF immediately after injury did not increase the number of BrdU+ cells [9]. The discrepancy might be explained by more effective and stable expression of VEGF by the *ex vivo* approach taken in this study.

Transplantation of F3.VEGF cells markedly increased the extent of proliferation of NG2+ glial progenitor cells. NG2 and PDGFα are coexpressed in O2A glial progenitor cells [28], and there is evidence that NG2+ cells differentiate into mature oligodendrocytes *in vitro* [29]. NG2 positive cells increase the extent of proliferation after SCI [13], and proliferating NG2 positive cells are thought to differentiate into mature oligodendrocytes replacing the ones that are lost secondary to injury [14]. We found that *ex vivo* delivery of VEGF increased the number of early proliferating cells that differentiated into mature oligodendrocytes at 6 weeks after SCI. These results suggest that VEGF expanded a pool of proliferating NG2 positive cells after SCI that eventually differentiated into mature oligodendrocytes. Notably, the number of newly born astrocytes at 6 weeks after SCI was not affected by F3.VEGF grafts. NG2+ cells could generate at least a portion of GFAP+ astrocytes composing the glial scar [30]. Our data suggest that the increase in proliferating NG2+ cells by F3.VEGF grafts did not contribute to the generation of new astrocytes in the glial scar. It is possible that proliferating NG2+ cells which are destined to take the astrocytic fate did not respond to VEGF. Alternatively, the effects of VEGF on genesis of new astrocytes might be antagonized by transplanted stem or progenitor cells as cellular vehicles that were shown to suppress activation of astrocytic scars [31], [32]. We did not find evidence that new neurons were generated by any of the experimental intervention. The molecular environment of the spinal cord is predominantly gliogenic, but not conducive for the generation of new neurons [33], [34]. We assumed that VEGF provided by F3.VEGF grafts could not overcome the limitation imposed by the molecular niches in the spinal cord.

Increases in the number of proliferating NG2+ glial progenitor cells and newly born oligodendrocytes can have important implications in the recovery of neurological function after SCI. Demyelinating lesions in the white matter are at least partially responsible for functional deficits after SCI [35], [36]. It has been demonstrated that newly generated oligodendrocytes are capable of remyelinating axons [16]. Therefore, it is conceivable that the expanded pool of proliferating glial progenitor cells by F3.VEGF could induce remyelination and lead to functional recovery. We showed that F3.VEGF grafts markedly enhanced tissue sparing. NG2 glial progenitor cells stimulated by VEGF might differentiate into myelinating oligodendrocytes and enhance remyelination, eventually leading to the increase in the volume of myelinated white matter. It is also possible that neuroprotective effects and/or angiogenic activity of VEGF played a more major role in enhanced tissue sparing. A single injection of VEGF immediately after SCI increased the density of blood vessels and decreased apoptosis of neural cells [9]. Moreover, VEGF also prevented retraction and promoted regeneration of the corticospinal axons after spinal cord transection [37]. Therefore, enhanced tissue sparing and the resulting improvement of locomotor function by F3.VEGF could be ascribed to a combination of the multifaceted trophic effects of VEGF on glial progenitor cells, endothelial cells, neural cells, and injured axons.

In summary, we showed a successful delivery of VEGF to the injured spinal cord tissue by genetically modified human NSCs as cellular vehicles. VEGF overexpressing NSC graft exerted a previously unreported effect of VEGF on glial progenitor cells following SCI: transplantation of F3.VEGF increased the proliferation of NG2+ glial progenitor cells and the number of newly born oligodendrocytes. Therefore, VEGF can be therapeutically utilized to repair white matter pathology in SCI. VEGF also improved angiogenesis and tissue sparing, indicating multifaceted actions for repair of injured spinal cord. The strategy of human NSC-based VEGF delivery may have potential to be clinically translated for the victims of SCI.

## Materials and Methods

### Preparation of VEGF overexpressing human NSCs

Telencephalon tissue from a 15 weeks gestational human fetal brain was utilized to generate primary cell culture from which immortalized cell lines of human NSCs (F3 line) were generated using a retroviral vector encoding v-myc oncogene. The permission to use the fetal tissues was granted by the Clinical Research Screening Committee involving Human Subjects of the University of British Columbia, and the fetal tissues were obtained from the Anatomical Pathology Department of Vancouver General Hospital. PG13 mouse packaging cell line was transfected with plasmid pLPCX-VEGF vector containing the full length human VEGF cDNA, using Lipofectamine 2000 (Invitrogen, Carlsbed, CA), and stable PG13 cell line was selected using 10 ug/ml puromycin for 3 days. Replication incompetent retroviral vector which was collected from PG13.VEGF cells was used for transfection of F3 human NSCs. Puromycin-resistant VEGF clones were screened and isolated, and one of the clones (F3.VEGF line) was expanded and used for the transplantation [38].

### Animals and surgical procedures

Adult Sprague–Dawley female rats, weighing 200–250 gm, were used in this study. Animal handling and surgical protocols followed the regulations set by the Ajou University Institutional Animal Care and Use Committee. After anesthetization with chloral hydrate (400 mg/kg, i.p.), animals received a dorsal laminectomy at the ninth thoracic vertebral level (T9) to expose the spinal cord, and then were subjected to mechanical impact with 180 kdyn force using the Infinite Horizon impactor (Precision Systems and Instrumentation, Lexington, KY). Seven days after injury, rats were randomly classified into three groups, and each group received injections of PBS, F3, and F3.VEGF cells, respectively. A total of 2×10^5^ cells, divided in two dosages, were transplanted into injured spinal cord. Each injections was made at 2 mm rostral and 2 mm caudal from the lesion epicenter, respectively, at a depth of 1.2 mm. At each site, 2 ul of cell suspension containing 10^5^ NSCs or vehicle was injected through a glass micropipette with a tip diameter of less than 60 um at a rate of 0.4 ul/min. All animals received daily intraperitoneal cyclosporine (Sandimmun; Novartis, Bern, Switzerland) at a dosage of 10 mg/kg beginning from one day prior to transplantation to three weeks after transplantation. After that, cyclosporine was administered through drinking water (50 µg/ml) until sacrifice.

### Bromodeoxyuridine (BrdU) injection

Cellular proliferation was examined using BrdU (Sigma, St. Louis, MO). To study the extent of cellular proliferation within 7 days after transplantation, animals were intraperitoneally injected with BrdU (50 mg/kg) for three consecutive days (4th, 5th, and 6th day), and then sacrificed the day after the last injection. To examine phenotypic fate of early proliferating cells, animals were injected with BrdU and were allowed to survive until the 35th day after transplantation, and then were sacrificed for histological analysis.

### Western blot analysis and ELISA

Spinal cord tissues spanning±5 mm from the epicenter were dissected and homogenized in ice-cold lysis buffer containing the followings: 20 mM Tris-HCl (pH 7.5), 1 mM EDTA, 5 mM MgCl_2_, 1 mM dithiothreitol, 0.1 mM phenylmethylsulfonyl fluoride, and protease inhibitor cocktail (Pierce, Rockford, IL). The tissue homogenate was centrifuged at 4°C and 14,000 rpm for 20 min, and protein concentration of the supernatant was measured using Bradford assay. Equal amounts of proteins were resolved by PAGE and transferred to a PVDF membrane (Millipore, Bedford, MA). The membrane was blocked in Tris buffered saline (TBS) containing 2.0% BSA and probed with the rabbit anti-flk-1 (1∶2000; Thermo Scientific, Fremont, CA) and rabbit anti-phospho-flk-1 (1∶2000; Millipore Corporation, Billerica, MA). After washing, the membranes were incubated for 1 hr at room temperature with secondary antibodies (1∶2000; Amersham Biosciences, Arlington Heights, IL). Finally, the blots were developed with enhanced chemiluminescence detection reagents (Amersham Biosciences, Arlington Heights, IL). The blots were reprobed with antibodies against mouse anti-β actin (1∶5000; Abcam, Cambridge, UK). Concentration of VEGF protein in injured spinal cord tissue was measured using ELISA (R&D systems, Minneapolis, MN) following an instruction manual provided by the manufacturer.

### Tissue processing and immunohistochemistry

Animals were deeply anesthetized with chloral hydrate and perfused intracardially with PBS, followed by 4% paraformaldehyde in 0.1 M phosphate buffer, pH 7.4. Spinal cords were removed and post-fixed in 4% paraformaldehyde for 2 hrs, followed by immersion into a graded series of sucrose solution. Cryosections of spinal cord were made transversely (section thickness 20 µm) in a 1∶10 series, and mounted onto SuperFrost Plus slides (Fisher Scientific, Pittsburgh, PA). For BrdU immunohistochemistry, transverse spinal cord sections were treated with 10% hydrogen peroxide to quench endogenous peroxidase activity, followed by incubation in 1 N HCl at 37°C for 30 min. After thorough rinsing and blocking, tissue sections were incubated with rat polyclonal anti-BrdU (1∶500; Serotec, Oxford, UK) at 37°C for 1 hour in a humid chamber. Tissue sections were rinsed and incubated with biotinylated horse anti-rat IgG secondary antibody, and the antigen-antibody reaction was visualized by Vectastain Elite ABC kit (Vector Laboratories, Burlingame, CA) with diaminobenzidine (DAB) substrate (Sigma-Aldrich, St. Louis, MO). For double-labeling with anti-BrdU and various cellular markers, transverse spinal cord sections were processed for BrdU staining as above and then extensively rinsed in PBS-T. Sections were blocked with the Avidin/Biotin blocking Kit (Vector Laboratories, Burlingame, CA) and incubated overnight with the second primary antibodies. After incubation with appropriate biotinylated secondary antibodies, sections were further processed using Vectastain Elite ABC kit with Vector SG peroxidase substrate kit (Vector Laboratories, Burlingame, CA). Development of antigen-antibody reactions with DAB substrate resulted in brown colored precipitates, whereas SG substrate yields bluish color, thus making it possible to identify two different cellular markers in the same section. For immunofluorescence staining, sections were incubated overnight with primary antibodies at 4°C or for 2 hrs at room temperature, followed by appropriate secondary antibodies tagged with Alexa Fluor 488 or Alexa Fluor 594 (Molecular Probes, Eugene, OR) for 45 min at room temperature. We used polyclonal NG2 antibody (1∶2000; Millipore, Bedford, MA) as a marker for oligodendrocyte progenitors, monoclonal CC1 antibody (1∶100; Calbiochem, La Jolla, CA) which recognizes adenomatous polyposis coli protein in cell bodies of mature oligodendrocyte without labeling of oligodendrocytic processes [39], [40], and polyclonal GFAP antibody (1∶500; Dako, Carpinteria, CA) for mature astrocytes. Primary antibodies were anti-BrdU, anti-GFAP, anti-CC1, monoclonal anti-human specific mitochondria (1∶500; Chemicon, Temecula, CA), anti-NeuN (1∶500; Chemicon, Temecula, CA), anti-MAP2 (1∶500; Chemicon, Temecula, CA), and polyclonal anti-von Willebrand Factor (vWF) (1∶250, Chemicon, Temecula, CA) which recognizes endothelial cells that produce vWF proteins.

### Stereological cell counts

The number of cells of interest was quantified using the unbiased stereological estimation, based on the optical fractionator method [41]. The Computer-Assisted Stereological Toolbox system, version 2.1.4 (Olympus, Ballerup, Denmark) equipped with an Olympus BX51 microscope, a motorized microscope stage (Prior Scientific, Rockland, MA) run by an IBM-compatible computer, and a microcastor (ND 281B, Heidenhain, Traunreut, Germany) connected to the stage and feeding the computer with the distance information in the *z*-axis was used. Using the injury epicenter as a point of reference, every third section extending rostral and caudal to the epicenter (sections spaced 200 um apart) was sampled so that measurements spanned a total of 6 mm (11 sections per animal, including the epicenter). The injured spinal cord was delineated at a 1.25× objective and generated counting grid of 300×300 um. An unbiased counting frame of known area superimposed on the image was placed randomly on the first counting area and systemically moved through all counting areas until the entire delineated area was sampled. Actual counting was performed using a 100× oil objective. The total number of cells was divided by spared tissue volume measured as described below to obtain the number of cells per 1 mm^3^ of spared tissue. Since the volume of spared spinal cord tissue was different between the groups, all the comparisons of the cell numbers between different groups were based on the density (i.e. the number of cells per 1 mm^3^ of spared spinal cord tissue) rather than absolute number. The number of cells at different regions from the epicenter was counted at the corresponding sections, and then was divided by the subvolume of the corresponding regions.

### Quantification of spinal cord tissue volume and microvessel density

To quantify the amount of white matter spared after SCI, sections surrounding the injury epicenter were stained with myelin specific eriochrome-cyanine RC [42]. Digitized photographs of the stained sections were taken using Olympus DP71 microscopy camera (Olympus, Ballerup, Denmark). The volume of spared spinal cord tissue was analyzed by the method of Cavalieri's principle [43]. The total cross-sectional areas of the spinal cord and the areas of cavity were stereologically measured using the Castor system. The areas of spared tissue were calculated by subtracting the cavity areas from the total cross sectional areas. The individual subvolumes of spared spinal cord tissue were obtained by multiplying the cross-sectional areas of spared tissue by the distance between sections, and the subvolumes were added to obtain the total volume of spared spinal cord tissue. Endothelial cell marker vWF stained sections were scanned at low power to determine the areas of highest vascular density. Within this region, 3 rectangular fields of interest were randomly selected at 20× objective lens. The areas occupied by vWF positive microvessels were measured using the publically available image J software (http://rsb.info.nih.gov/ij/index.html), and were divided by the total areas of the corresponding fields to obtain the density of microvessels in the injured spinal cord tissue

### Analysis of locomotor behavior

The recovery of gross overground locomotion was evaluated within 24 hrs after an initial injury, within 24 hrs after transplantation or vehicle injection and once a week thereafter using the Basso, Beattie, and Bresnahan (BBB) open field locomotor test [44]. After transplantation, animals were assigned a randomized code to ensure blind evaluation of locomotor performance (PBS N = 8, F3 N = 8, F3.VEGF N = 8). BBB score was determined 7 times; within 24 hrs after an initial injury, within 24 hrs after transplantation or vehicle injection, and then once a week until 6 weeks after injury. Its scale ranges from 0 to 21, with 0 given to animals with no observable hindlimb movement and 21 given to animals with completely normal open field locomotor function.

### Statistical analysis

All statistical analyses were performed using the PRISM 4.0 statistical software package (Graphpad, Inc., San Diego, CA). Differences among experimental groups were evaluated by a one-way ANOVA followed by a Tukey's *posthoc* analysis. Repeated measures two-way ANOVA was used to compare matched data at multiple time points. Significance for all statistical analysis was set at *p<0.05*. Error bars in all graphs depict standard deviation (SD) of measured variables.

## References

[1] Schwab JM, Brechtel K, Mueller CA, Failli V, Kaps HP (2006). Experimental strategies to promote spinal cord regeneration–an integrative perspective.. Prog Neurobiol.

[2] Blesch A, Lu P, Tuszynski MH (2002). Neurotrophic factors, gene therapy, and neural stem cells for spinal cord repair.. Brain Res Bull.

[3] Rosenstein JM, Krum JM (2004). New roles for VEGF in nervous tissue–beyond blood vessels.. Exp Neurol.

[4] Jin KL, Mao XO, Greenberg DA (2000). Vascular endothelial growth factor: direct neuroprotective effect in in vitro ischemia.. Proc Natl Acad Sci U S A.

[5] Matsuzaki H, Tamatani M, Yamaguchi A, Namikawa K, Kiyama H (2001). Vascular endothelial growth factor rescues hippocampal neurons from glutamate-induced toxicity: signal transduction cascades.. FASEB J.

[6] Khaibullina AA, Rosenstein JM, Krum JM (2004). Vascular endothelial growth factor promotes neurite maturation in primary CNS neuronal cultures.. Brain Res Dev Brain Res.

[7] Mani N, Khaibullina A, Krum JM, Rosenstein JM (2005). Astrocyte growth effects of vascular endothelial growth factor (VEGF) application to perinatal neocortical explants: receptor mediation and signal transduction pathways.. Exp Neurol.

[8] Sondell M, Lundborg G, Kanje M (1999). Vascular endothelial growth factor has neurotrophic activity and stimulates axonal outgrowth, enhancing cell survival and Schwann cell proliferation in the peripheral nervous system.. J Neurosci.

[9] Widenfalk J, Lipson A, Jubran M, Hofstetter C, Ebendal T (2003). Vascular endothelial growth factor improves functional outcome and decreases secondary degeneration in experimental spinal cord contusion injury.. Neuroscience.

[10] Storkebaum E, Lambrechts D, Dewerchin M, Moreno-Murciano MP, Appelmans S (2005). Treatment of motoneuron degeneration by intracerebroventricular delivery of VEGF in a rat model of ALS.. Nat Neurosci.

[11] Sun Y, Jin K, Xie L, Childs J, Mao XO (2003). VEGF-induced neuroprotection, neurogenesis, and angiogenesis after focal cerebral ischemia.. J Clin Invest.

[12] Weiss S, Dunne C, Hewson J, Wohl C, Wheatley M (1996). Multipotent CNS stem cells are present in the adult mammalian spinal cord and ventricular neuroaxis.. J Neurosci.

[13] McTigue DM, Wei P, Stokes BT (2001). Proliferation of NG2-positive cells and altered oligodendrocyte numbers in the contused rat spinal cord.. J Neurosci.

[14] Zai LJ, Wrathall JR (2005). Cell proliferation and replacement following contusive spinal cord injury.. Glia.

[15] Tripathi R, McTigue DM (2007). Prominent oligodendrocyte genesis along the border of spinal contusion lesions.. Glia.

[16] Yang H, Lu P, McKay HM, Bernot T, Keirstead H (2006). Endogenous neurogenesis replaces oligodendrocytes and astrocytes after primate spinal cord injury.. J Neurosci.

[17] Jin K, Zhu Y, Sun Y, Mao XO, Xie L (2002). Vascular endothelial growth factor (VEGF) stimulates neurogenesis in vitro and in vivo.. Proc Natl Acad Sci U S A.

[18] Behrstock S, Ebert A, McHugh J, Vosberg S, Moore J (2006). Human neural progenitors deliver glial cell line-derived neurotrophic factor to parkinsonian rodents and aged primates.. Gene Ther.

[19] Park KI, Himes BT, Stieg PE, Tessler A, Fischer I (2006). Neural stem cells may be uniquely suited for combined gene therapy and cell replacement: Evidence from engraftment of Neurotrophin-3-expressing stem cells in hypoxic-ischemic brain injury.. Exp Neurol.

[20] Martinez-Serrano A, Bjorklund A (1998). Ex vivo nerve growth factor gene transfer to the basal forebrain in presymptomatic middle-aged rats prevents the development of cholinergic neuron atrophy and cognitive impairment during aging.. Proc Natl Acad Sci U S A.

[21] Jandial R, Singec I, Ames CP, Snyder EY (2008). Genetic modification of neural stem cells.. Mol Ther.

[22] Ferrara N, Gerber HP, LeCouter J (2003). The biology of VEGF and its receptors.. Nat Med.

[23] Breakefield X, Jacobs A, Wang S, Tuszynski MH, Kordower J (1999). Genetic engineering for CNS regeneration.. CNS regeneration.

[24] Muller FJ, Snyder EY, Loring JF (2006). Gene therapy: can neural stem cells deliver?. Nat Rev Neurosci.

[25] Namiki J, Tator CH (1999). Cell proliferation and nestin expression in the ependyma of the adult rat spinal cord after injury.. J Neuropathol Exp Neurol.

[26] Yourey PA, Gohari S, Su JL, Alderson RF (2000). Vascular endothelial cell growth factors promote the in vitro development of rat photoreceptor cells.. J Neurosci.

[27] Rosenstein JM, Mani N, Silverman WF, Krum JM (1998). Patterns of brain angiogenesis after vascular endothelial growth factor administration in vitro and in vivo.. Proc Natl Acad Sci U S A.

[28] Nishiyama A, Lin XH, Giese N, Heldin CH, Stallcup WB (1996). Co-localization of NG2 proteoglycan and PDGF alpha-receptor on O2A progenitor cells in the developing rat brain.. J Neurosci Res.

[29] Nishiyama A, Chang A, Trapp BD (1999). NG2+ glial cells: a novel glial cell population in the adult brain.. J Neuropathol Exp Neurol.

[30] Alonso G (2005). NG2 proteoglycan-expressing cells of the adult rat brain: possible involvement in the formation of glial scar astrocytes following stab wound.. Glia.

[31] Li Y, Chen J, Zhang CL, Wang L, Lu D (2005). Gliosis and brain remodeling after treatment of stroke in rats with marrow stromal cells.. Glia.

[32] Davies JE, Huang C, Proschel C, Noble M, Mayer-Proschel M (2006). Astrocytes derived from glial-restricted precursors promote spinal cord repair.. J Biol.

[33] Shihabuddin LS, Horner PJ, Ray J, Gage FH (2000). Adult Spinal Cord Stem Cells Generate Neurons after Transplantation in the Adult Dentate Gyrus.. J Neurosci.

[34] Horner PJ, Power AE, Kempermann G, Kuhn HG, Palmer TD (2000). Proliferation and differentiation of progenitor cells throughout the intact adult rat spinal cord.. J Neurosci.

[35] Waxman SG (1992). Demyelination in spinal cord injury and multiple sclerosis: what can we do to enhance functional recovery?. J Neurotrauma.

[36] Cao Q, Zhang YP, Iannotti C, DeVries WH, Xu XM (2005). Functional and electrophysiological changes after graded traumatic spinal cord injury in adult rat.. Exp Neurol.

[37] Facchiano F, Fernandez E, Mancarella S, Maira G, Miscusi M (2002). Promotion of regeneration of corticospinal tract axons in rats with recombinant vascular endothelial growth factor alone and combined with adenovirus coding for this factor.. J Neurosurg.

[38] Lee HJ, Kim KS, Park IH, Kim SU (2007). Human neural stem cells over-expressing VEGF provide neuroprotection, angiogenesis and functional recovery in mouse stroke model.. PLoS ONE.

[39] Rabchevsky AG, Sullivan PG, Scheff SW (2007). Temporal-spatial dynamics in oligodendrocyte and glial progenitor cell numbers throughout ventrolateral white matter following contusion spinal cord injury.. Glia.

[40] Bhat RV, Axt KJ, Fosnaugh JS, Smith KJ, Johnson KA (1996). Expression of the APC tumor suppressor protein in oligodendroglia.. Glia.

[41] West MJ, Slomianka L, Gundersen HJ (1991). Unbiased stereological estimation of the total number of neurons in thesubdivisions of the rat hippocampus using the optical fractionator.. Anat Rec.

[42] Rabchevsky AG, Fugaccia I, Turner AF, Blades DA, Mattson MP (2000). Basic fibroblast growth factor (bFGF) enhances functional recovery following severe spinal cord injury to the rat.. Exp Neurol.

[43] Gundersen G, Andreassen HP (1998). Causes and consequences of natal dispersal in root voles, Microtus oeconomus.. Anim Behav.

[44] Basso DM, Beattie MS, Bresnahan JC (1995). A sensitive and reliable locomotor rating scale for open field testing in rats.. J Neurotrauma.

